# Associative nitrogen fixation (ANF) in switchgrass (*Panicum virgatum*) across a nitrogen input gradient

**DOI:** 10.1371/journal.pone.0197320

**Published:** 2018-06-01

**Authors:** Sarah S. Roley, David S. Duncan, Di Liang, Aaron Garoutte, Randall D. Jackson, James M. Tiedje, G. Philip Robertson

**Affiliations:** 1 WK Kellogg Biological Station, Michigan State University, Hickory Corners, Michigan, United States of America; 2 Great Lakes Bioenergy Research Center, Michigan State University, East Lansing, Michigan, United States of America; 3 Department of Agronomy, University of Wisconsin-Madison, Madison, Wisconsin, United States of America; 4 Great Lakes Bioenergy Research Center, University of Wisconsin-Madison, Madison, Wisconsin, United States of America; 5 Department of Plant, Soil and Microbial Sciences, Michigan State University, East Lansing, Michigan, United States of America; 6 Center for Microbial Ecology, Michigan State University, East Lansing, MI, United States of America; USDA Agricultural Research Service, UNITED STATES

## Abstract

Associative N fixation (ANF), the process by which dinitrogen gas is converted to ammonia by bacteria in casual association with plants, has not been well-studied in temperate ecosystems. We examined the ANF potential of switchgrass (*Panicum virgatum L*.), a North American prairie grass whose productivity is often unresponsive to N fertilizer addition, via separate short-term ^15^N_2_ incubations of rhizosphere soils and excised roots four times during the growing season. Measurements occurred along N fertilization gradients at two sites with contrasting soil fertility (Wisconsin, USA Mollisols and Michigan, USA Alfisols). In general, we found that ANF potentials declined with long-term N addition, corresponding with increased soil N availability. Although we hypothesized that ANF potential would track plant N demand through the growing season, the highest root fixation rates occurred after plants senesced, suggesting that root diazotrophs exploit carbon (C) released during senescence, as C is translocated from aboveground tissues to roots for wintertime storage. Measured ANF potentials, coupled with mass balance calculations, suggest that ANF appears to be an important source of N to unfertilized switchgrass, and, by extension, to temperate grasslands in general.

## Introduction

Nitrogen (N) sometimes accumulates in temperate terrestrial ecosystems beyond what can be explained by known inputs from atmospheric deposition, fertilizer addition, and biological nitrogen fixation (BNF) by legumes and actinorhizal plants [1–3]. These unexplained inputs are often hypothesized to be from BNF by microbes living outside of root nodules. However, BNF has not been well-studied in ecosystems that lack nodulating plants and it is unclear whether BNF rates are sufficient to account for the extra N accumulation.

BNF, the microbial conversion of dinitrogen gas (N_2_) to ammonia (NH_3_), is a high-energy process, with 16 ATP required to fix 1 molecule of N_2_ [4]. The high energy requirements are often met via a plant-microbe mutualism, in which plants provide microbes with fixed carbon (C) and an oxygen-controlled environment via root nodules [5]. But N fixation can also occur by free-living microbes and by microbes associated with non-nodulating plants. In free-living fixation, organisms fix N_2_ without external partners, whereas in associative N fixation (ANF), there is a casual association between plants and diazotrophs, but the two partners lack lasting interdependence [5]. Free-living fixation and ANF are hard to distinguish from one another in the rhizosphere [6]; here, we refer to any N fixation in the plant rhizosphere as ANF, but acknowledge that some of the fixation may occur by free-living diazotrophs. ANF has been long-documented in sugarcane (*Saccharum officinarum*) [7] and other tropical grasses [8, 9] where N-fixing microbes are either closely associated with root surfaces [10] or are endophytic [11]. Although ANF occurs in a number of tropical and temperate species, much uncertainty remains about its contribution to plant and ecosystem N budgets, seasonal changes in its rates, and its response to external N inputs, especially in temperate regions [12, 13].

Switchgrass (*Panicum virgatum* L.), a C4 grass historically dominant in North American prairies and savannas [14], is a potential model organism for ANF in temperate regions. Switchgrass has been well-studied for its use as a forage grass and more recently for its potential as a cellulosic biofuel [15, 16]. In agronomic settings, the yields of switchgrass often do not respond to N inputs and remain consistently high even as annual harvest removes N [17, 18]. In the absence of substantial N deposition or soil organic N depletion, the only logical source of replacement N is ANF. Indeed, switchgrass can incorporate recently fixed N into its tissues [19, 20], and diverse communities of N-fixing bacteria are present in switchgrass rhizospheres [21, 22], a prerequisite for ANF.

To explore the potential for ANF in switchgrass rhizospheres, we measured ANF potential in switchgrass plots throughout the growing season under a range of N input levels. Because of its high energy requirements, BNF rates typically increase as soil N becomes limiting (e.g., [23–25]) and thus we expected ANF rates to decrease with N fertilizer inputs. Similarly, we predict that ANF rates will coincide with plant N demand and consequent depletion of N in the rhizosphere [20, 26, 27].

We also explore the role of soil organic N in the switchgrass budget by measuring soil N mineralization rates. Soil N mineralization typically increases with soil N (e.g., [28, 29]), but at a particular soil N level, N mineralization exhibits seasonal signals that correspond with plant growth. N mineralization rates tend to increase through the growing season, peaking in June or July and then decreasing through the late summer and autumn [30]. Thus, we expected that ANF and N mineralization would have opposite responses to soil N (ANF a negative relationship; mineralization a positive relationship), but similar seasonal signals. To evaluate the generality of these responses, we conducted this study at two sites with different capacities to supply soil N.

Our goal was to use seasonal ANF potential measurements across N fertilization levels and soil types to evaluate and refine the hypothesis that ANF contributes a substantial portion of the N needed by temperate, perennial grasses. In doing so, we tested the following specific hypotheses: 1) ANF potentials decline with N fertilizer additions that inhibit ANF, and 2) ANF potentials track plant N demand through the growing season, coincident with seasonal changes in soil N.

## Methods

### Site description

We conducted our measurements along a switchgrass N fertilization gradient at two field sites of the Great Lakes Bioenergy Research Center (GLBRC), both positioned in the northern part of the US Corn Belt. One site is located at the Kellogg Biological Station Long-Term Ecological Research Site (lter.kbs.msu.edu) in southwestern Michigan, USA (42° 23’N 85°22’W, elevation 284 m asl). The other site is located at the Arlington Agricultural Research Station (arlington.ars.wisc.edu/) in south central Wisconsin, USA (43° 8’N, 89°21’N, elevation 315 m asl). Both sites have temperate climates, with the Michigan site (MI) receiving 1005 mm annual precipitation and having an annual average temperature of 10.1° C [31], and the Wisconsin site (WI) receiving 869 mm annual precipitation and having an annual average temperature of 6.8° C [32]. The soils at MI are Alfisol loams (Kalamazoo series Typic Hapludalfs) formed from glacial outwash [33], while the soils at WI are Mollisol silt loams (Plano series Typic Argiudolls), developed from deep loess deposits [34]. Both sites were cropped for decades (including maize, soybeans, and alfalfa) prior to switchgrass establishment.

At both sites, switchgrass (Cave-in-rock variety) was seeded in the summer of 2008. At MI, N treatments were established in 2009 and included four blocks, each containing eight 4.6 m × 7.6 m plots, each receiving one of eight fertilizer treatments, ranging from 0 to 196 kg N ha^-1^ yr^-1^ (https://lter.kbs.msu.edu/maps/images/current-switchgrass-nitrogen-experiment.pdf). At WI, N treatments were established in 2014, and included four blocks, each containing six 4.6 m x 10.7 m fertilizer treatments with the same N addition range as at MI. A randomized complete block design was used at both sites. The experiment was designed to test the response of switchgrass yields to N fertilizer addition and establish optimal fertilizer levels. We sampled three treatments: 0, 56, and 196 kg N ha^-1^ yr^-1^ (hereafter, 0-N, 56-N, and 196-N, respectively). The 0-N and 196-N treatments correspond to the lowest and highest Nin the experiment, while the 56-N treatment is the recommended agronomic rate that corresponds to the amount of N expected to be removed during harvest (the replacement value). No other fertilizer application occurred (e.g., no phosphorus or potassium were added).

At both sites, fertilizer application occurred in the spring by spraying liquid fertilizer from a tractor-mounted boom-type sprayer. At MI, N was applied as urea-ammonium-nitrate (28% N solution), and at WI, N was applied as ammonium nitrate (34% N solution), and the volume was scaled to achieve the N addition levels described above. Beginning in 2009, switchgrass was harvested once annually after the first killing frost, usually in October, for which the aboveground biomass was cut 15 cm above the soil surface with commercial forage harvesting equipment and weighed. A sub-sample was weighed, oven-dried at 60° to constant mass, and reweighed, and all yields were corrected to dry weight. To minimize edge effects, we recorded the yield only from a 2.3 m wide strip in the middle of the plot (i.e., 2.3 m × 7.6 m at MI; 2.3 m × 10.7 m at WI), but removed the aboveground biomass from the entire plot after yield determination.

### Nitrogen mass balance determination

In order to provide boundaries for annual estimates of ANF and to inform our hypotheses, we estimated the N deficit at the MI site with a mass balance determination that included N removed in harvest, atmospheric N deposition, and fertilizer N. This approach excludes losses from leaching and denitrification, which makes our N deficit estimate conservative. Furthermore, our purpose with the mass balance was not to delineate each component of the N cycle; rather, it was to provide a reasonable estimate of N fixation with which to compare our empirical estimates.

We ground the harvested biomass and measured total carbon (C) and N content by combustion on a Costech Elemental Combustion System 4010 (Valencia, CA). We multiplied the percent N by the harvested yield to determine the amount of N removed from the plots each year. We obtained annual atmospheric N deposition data–collected at MI–from the National Atmospheric Deposition Program (NADP; nadp.isws.illinois.edu). The annual N mass balance was calculated as N inputs from fertilizer plus atmospheric deposition, minus N removed during harvest. We did not have adequate data to complete the N mass balance at WI.

### Field root and soil sampling

We sampled 4 times in 2015, with each sampling period corresponding to a phenological stage: 1) May, after switchgrass emergence, but before fertilizer application; 2) June, 2 to 3 weeks after fertilizer addition; 3) late July, at peak biomass, when the switchgrass was starting to flower; and 4) October, after the switchgrass had senesced, but prior to harvest. During each sampling period, we collected soil samples from all four replicates of three fertilizer treatments: 0-N, 56-N, and 196-N.

During each sampling period, we removed a soil core (15 cm depth × 4.7 cm diameter) from each replicate plot with a hammer coring device (Forestry Suppliers, Jackson, MS). Each core was taken 3 to 5 cm from a switchgrass crown. We extruded the core, passed it through a 4-mm diameter sieve, removed visible roots, and refrigerated the soil until the following day. The sieved soil was used to measure net N mineralization, net nitrification, and ANF rates as described below. The visible roots were saved from each core, washed with unchlorinated groundwater to remove all visible soil, and used to assay root ANF.

### Nitrogen fixation potential

We measured ANF potential using ^15^N_2_ in vitro incubations of soils and roots [35]. After sieving, we measured 5 g of soil from each treatment plot into each of two 12-ml Exetainers (Labco, Lampeter, Ceredigion, UK). We left the soil in the Exetainers, loosely capped, for 1 to 2 d to equilibrate. We then added enough 4% glucose solution (w/v) to raise the water-filled pore space (WFPS, [36]) to 100% and to compensate for any evaporation that occurred during the equilibration period. The amount of glucose solution added ranged from 0.9 mL to 1.9 mL, which is equivalent to 7.2 to 15.2 mg C per sample, enough to saturate the C demands of N fixers [35]. For root samples, we added 1 g of coarse roots to each of two vials. We sometimes had to combine roots from two replicate plots to obtain sufficient material, but always had samples from at least 3 replicate plots per treatment and site. We added enough 4% glucose solution to the roots to replace any water loss from evaporation that occurred between weighing the roots into Exetainers and starting the incubation (0.08 to 0.2 mL of solution containing 0.6 mg C to 1.6 mg C).

In separating roots from the plant, we created a conservative N fixation environment insofar as endophytic bacteria would have less access to plant materials and surface-dwelling bacteria would have less access to newly exuded photosynthate. The glucose addition was designed to relieve C demands so created. We also note that adding C to excised roots did not stimulate fixation (S1 Text, S1–S3 Figs), suggesting that the diazotrophs on or in the roots were not C-limited.

We tightly capped each Exetainer and then removed 4 ml of headspace air with a syringe. We added 4.2 ml of either ultra-high purity N_2_ (controls) or ^15^N_2_ gas, enriched to 99% (Cambridge Isotope Laboratories, Tewksbury, MA, USA), which resulted in ^15^N_2_ concentration of ~50% in the Exetainer. We stored the samples in the dark at room temperature for 7 d. We then opened the Exetainers and measured the headspace volume by water displacement and placed the contents of the Exetainers into aluminum pans in the drying oven at 60°C. Once the samples had dried to constant temperature, they were weighed, ground to a fine powder in a SPEX Shatterbox (Metuchen, NJ, USA), and packed in tin capsules for isotopic analysis (University of California, Davis Stable Isotope Facility, USA).

We calculated ANF, in μg N vial^-1^ d^-1^, as

$$\frac{\left({AE}_{i}\times {TN}_{i}\right)}{\left({AE}_{atm}\times t\right)}$$ (1)

where TN_i_ is the total amount of N in the soil or root, t is incubation time, AE_i_ is the atom excess in the soil or roots, calculated as the difference in ^15^N atom% of the soil or roots between the treatment vial and control vial [37]. We calculated the atom excess in the headspace (AE_atm_) by dividing the volume of ^15^N_2_ added by the total N_2_ in the headspace, assuming atmospheric concentration of ^14^N_2_. Our AE_atm_ calculations may be slight overestimates, because they did not account for potential changes in N_2_ concentration in the vial as a result of denitrification. These effects were likely small, though, and the result of an overestimated AE_atm_ is a lower ANF rate, making our estimates conservative. We then divided each rate by the dry mass of the material in the Exetainer, resulting in N_2_ fixation expressed as μg N g^-1^ d^-1^.

### Assessing the effects of laboratory conditions on nitrogen fixation estimates

Laboratory ANF potential assays are easier and less expensive than whole-plant or field assays, which allow for more treatments and replicates, but they create some artifacts. Two of our assay conditions may have inflated ANF estimates: 1) glucose addition and 2) low oxygen concentration. High C availability and low oxygen both promote N fixation [38], and an environment optimized for N fixation may favor the growth of diazotrophs. We thus conducted a series of experiments to quantify the effect of these conditions on our rate estimates. These experiments are detailed in the Supplemental information (S1 Text, S1–S3 Figs). We found that in soils, the addition of glucose solution stimulated ANF by a factor of 4.8, on average (S1 Fig). Glucose only stimulated ANF when added as solution; neither deionized water, powdered glucose, nor anaerobic headspace conditions stimulated ANF (S2 Fig). In roots, addition of glucose in solution did not stimulate ANF (S3 Fig). In summary, the in vitro technique is appropriate for comparison among treatments, but to scale the measurements, we must first account for the effect of glucose addition.

### Contamination checks on ^15^N_2_

Commercially-available ^15^N_2_ can be contaminated with other forms of reactive ^15^N, including ^15^NH_3_ and ^15^NO_x_^-^, that readily oxidize to ^15^NO_3_^-^ when dissolved in water [39]. These N forms are readily taken up by plants and microbes, which can result in ^15^N enrichment in the absence of fixation and thus inflate BNF estimates or result in false positives. We tested for contamination of our source ^15^N_2_ (from Cambridge Isotope Laboratories, Tewksbury, MA, USA) and also calculated the effect of potential contaminants on ANF rates. We found no evidence of contamination, as detailed in the Supplemental information (S2 Text).

### Estimates of annual nitrogen inputs from fixation

In addition to mass balance-based estimates, we calculated a first-order approximation of annual ANF by extrapolating our measured ANF rates. While imperfect, extrapolation likely provides an upper boundary for system-wide ANF rates. For this estimate, we multiplied soil fixation (unit: μg N g^-1^ soil d^-1^) by the mean bulk density and core depth from each site. Bulk density was determined from previous soil coring efforts, in which a hydraulic corer was used to collect soil and minimize compaction. To correct for the effect of glucose addition, we divided the ANF rates by 4.8 (the factor by which glucose increased N_2_ fixation in the unfertilized plots, S2 Fig). We assumed a growing season of 120 d (~May 1 to Aug 29) and partitioned fixation rates as follows: pre-fertilization 20 d, post-fertilization 40 d, peak biomass 60 d.

We also scaled root ANF at MI by multiplying root ANF rates by areal estimates of root biomass in the surface 25 cm at MI (root biomass data were not available at WI). We assume consistent ANF rates in roots, regardless of depth. Because we observed no increase in ANF with C addition (S4 Fig), we used the rates as measured in our lab assays. We applied the same growing-season assumptions as with soil ANF.

Our intent in scaling our laboratory measurements is not to provide a definitive measure of annual ANF rates but rather to evaluate agreement between measures of ANF with mass balance estimates. If, for example, scaled laboratory measurements are substantially lower than our mass balance estimates, this calculation would suggest that it was not possible to make up the N deficit with ANF. In contrast, if the two estimates are of similar magnitude, it suggests that ANF has the potential to provide sufficient N to make up the deficit.

### Net potential mineralization and nitrification

We measured net potential N mineralization and nitrification in 28-d aerobic incubations [40]. After sieving, we measured gravimetric soil moisture and calculated WFPS based on known bulk density and soil porosity at each site. We then added deionized water to each soil, sufficient to raise the WFPS to 60%. In doing so, we optimized conditions for mineralization and nitrification; these are potential rates, rather than field rates, which provide good approximations of relative N availability [40]. From each soil core, we weighed 6 replicates of 10 g each into 150-ml polyethylene specimen cups. We extracted nitrate (NO_3_^-^) and ammonium (NH_4_^+^) (nitrate_T0_ and ammonium_T0_) from three cups by adding 100 mL of 1 M potassium chloride, shaking for 1 min, allowing to sit for 24 h, then shaking again and filtering the supernatant. Filtrate samples were placed in the freezer until analysis. We loosely capped the remaining three cups and stored in a dark cupboard at room temperature for 28 d. We maintained soil moisture by weighing all cups weekly and adding deionized water sufficient to replace water lost through evaporation. After 28 d, we extracted the NO_3_^-^ and NH_4_^+^ (nitrate_T28_ and ammonium_T28_) in the same way as at T0 and stored the extracts in the freezer until analysis.

We analyzed the extracts for NO_3_^—^N and NH_4_^+^-N concentration using the cadmium reduction and the phenolhypochlorite methods, respectively, using a Lachat QuikChem 8500 flow injection analyzer (Hach, Loveland, CO).

We calculated net mineralization as:

$$N_{mineralized}=\frac{\left({nitrate}_{T28}+{ammonium}_{T28})-({nitrate}_{T0}+{ammonium}_{T0}\right)}{28\;days}$$ (2)

We calculated net nitrification as:

$$N_{nitrified}=\frac{\left({nitrate}_{T28}-{nitrate}_{T0}\right)}{28\;days}$$ (3)

### Soil total carbon and nitrogen

We measured total C and N pools on soil collected from all treatments and sites at the post-senescence sampling time. We pulverized the soil (<0.5 mm) and measured the total C and total N content on a Costech Elemental Combustion System 4010 (Valencia, CA).

### Statistical analysis

To examine switchgrass yield response to N fertilizer addition, we used quadratic plateau models [41] weighted by the inverse of rank order [18], which allowed us to calculate the optimal N addition for each year of the experiment. The optimal N addition is the minimum amount of N fertilizer needed to yield the maximum biomass. To determine if N transformation rates varied with time and N fertilizer addition, we used a fixed effects multiple linear regression model to determine which parameters and interactions were statistically significant and which model best fit the data, based on Akaike Information Criterion (AIC).

The multiple linear regression approach allowed us to examine overall drivers of transformations, but we also sought to understand the nature of the effect of N addition (e.g., linear, exponential, or saturating (i.e., asymptotic)). To do so, we regressed each N transformation rate with N addition level within individual time periods (n = 4 x 3 fertilizer treatments = 12 data points for each regression). We fit a linear or non-linear line to the data, using gls (linear) or gnls (non-linear) in the mgcv package in R [42] and chose the statistically significant fit with the lowest AIC value. If none of the regression lines had statistically significant parameters, we concluded that there was no difference among N fertilizer treatments in that time period. If the data did not meet assumptions of normality and homogeneity, we employed alternate variance structures that accounted for these deviations [43].

To determine the drivers of mineralization and ANF, we used linear and non-linear regression with soil mineral N (NO_3_^-^, NH_4_^+^, and total inorganic N concentration) as predictors of N_2_ fixation and mineralization rates, using the methods described above to determine the significant predictors and the type of relationship. We used ANOVA and Tukey’s Honestly Significant Difference test to compare soil inorganic N concentrations among treatments. Finally, we used Welch’s paired t-test to determine the effect of glucose addition on soil fixation. We used R version 3.2.3 [44] for all analyses.

## Results

### Nitrogen mass balance

The switchgrass N yield response was variable through time. At MI, the optimal N fertilizer level declined each year of establishment, from 147 kg N ha^-1^ in 2009 to 0 kg N ha^-1^ in 2012 through 2014 (Table 1, [18]). Note that an optimal fertilizer value of 0 means that N fertilizer had no effect on yield. In 2015, yields again exhibited an N response, with an optimal N fertilizer level of 109 kg N ha^-1^ yr^-1^. Despite the decline in response to N addition, yields generally increased over the first four years and remained consistent in the following years. At WI, there was a strong fertilizer response in the first year (2014), with the optimal N level at 194 kg N ha^-1^ yr^-1^. The next year, optimal N declined to 90 kg N ha^-1^ yr^-1^.

**Table 1:** Nitrogen mass balance for switchgrass at the MI site.

			0-N^e^			56-N^e^			196-N^e^		
Year	Atm dep^a^	Optimal N^b^	Harvest yield^c^	N output^d^	N balance^f^	Harvest yield^c^	N output^d^	N balance^f^	Harvest yield^c^	N output^d^	N balance^f^
2009	5.2	147^g^	2.0 (0.2)	6.2 (0.5)	-1.0 (0.5)	3.6 (0.2)	16.0 (0.7)	45.3 (0.7)	4.3 (0.4)	28.8 (2.5)	172.6 (2.5)
2010	5.7	72 ^g^	6.2 (0.4)	19.0 (2.6)	-13.3 (2.6)	9.3 (1.2)	39.0 (1.8)	22.7 (1.8)	8.3 (0.7)	62.5 (5.7)	139.3 (5.7)
2011	8.0	34 ^g^	9.6 (0.2)	41.4 (3.8)	-33.4 (3.8)	10.6 (0.4)	68.7 (5.6)	-4.6 (5.6)	10.8 (1.1)	83.7 (7.3)	120.5 (7.3)
2012	5.6	0	7.2 (0.6)	31.1 (2.8)	-25.5 (2.8)	7.4 (0.2)	47.8 (1.2)	13.8 (1.2)	5.5 (0.2)	45.9 (2.0)	155.8 (2.0)
2013	7.3	0	11.9 (0.4)	70.4 (9.5)	-63.1 (9.5)	11.6 (0.7)	87.9 (7.8)	-24.6 (7.8)	10.7 (0.5)	101.6 (5.7)	101.8 (5.7)
2014	6.1	0	11.3 (0.7)	65.2 (4.3)	-59.1 (4.3)	11.8 (0.5)	84.4 (3.9)	-22.3 (3.9)	12.0 (0.4)	109.9 (3.6)	92.3 (3.6)
2015	5.6	109	10.9 (0.9)	56.7 (10.1)	-51.1 (10)	12.3 (0.1)	93.7 (3.5)	-32.0 (3.5)	12.9 (0.4)	92.9 (4.0)	108.9 (4.0)
Mean	6.2 (0.4)	52 (22)		41.4 (2.2)	-35.2 (2.2)		62.5 (1.6)	-0.24 (1.6)		75.0 (1.8)	127.3 (1.8)
Total					-246.5 (16)			-1.7 (11)			891.2 (13)

^a^Atm dep is the atmospheric deposition of N, in kg N ha^-1^ yr^-1^.

^b^Optimal N is the amount of N fertilizer needed to achieve maximum biomass, in kg N ha^-1^ yr^-1^. This was calculated with yield data from 8 fertilizer treatments (0, 28, 56, 84, 112, 140, 168, and 196 kg N ha^-1^ yr^-1^).

^c^Harvest yield is the total biomass removed in the harvest, in Mg switchgrass ha^-1^ yr^-1^.

^d^N output is the N removed in harvest and is reported as the mean of 4 replicates (standard error of the mean).

^e^0-N are unfertilized plots, 56-N are plots fertilized at 56 kg N ha^-1^ yr^-1^, and 196-N are plots fertilized at 196 kg N ha^-1^ yr^-1^.

^f^N balance is the inputs (fertilizer inputs plus atmospheric deposition) minus outputs, with a negative value indicating net loss of N (deficit).

^g^from [18]

In all years, the 0-N plots at MI exhibited an N deficit, with N removal during harvest exceeding inputs from atmospheric deposition. The deficit ranged from 1.0 ± 0.5 [SE] kg N ha^-1^ yr^-1^ (2009) to 63.1 ± 9.5 kg N ha^-1^ yr^-1^ (2013) and was 51.1 kg N ha^-1^ yr^-1^ in 2015. The 56-N plots also exhibited an N deficit in most years, despite receiving annual fertilizer inputs (Table 1). Over the 6-y course of the experiment, the 0-N and the 56-N plots had cumulative N deficits of 247 and 1.7 kg N ha^-1^, respectively, which equates to an average deficit of 35.2 ± 2.2 kg N ha^-1^ yr^-1^ in the 0-N plot (Table 1). During the post-establishment phase (2013–2015), the average deficit was 58 kg N ha^-1^ yr^-1^, an upper-bound mass balance estimate of annual fixation at this site.

### Soil nitrogen and carbon pools

Soil inorganic N concentrations in the three N treatments tracked N fertilizer addition levels. Prior to fertilizer addition each year, plots in the three treatments had similar soil N concentrations, which increased after N addition (Table 2). At MI, the fertilizer effect in the 56-N and 196-N treatments was no longer present by peak biomass, but reappeared in the 196-N treatment after plant senescence. At WI, the fertilizer effect remained through peak biomass, but diminished by plant senescence (Table 2).

**Table 2:** Inorganic N concentrations by site and sampling time^a^.

		Pre-fertilizer (May)		Post-fertilizer (June)		Peak biomass (July)		Senescence (October)	
Site	N addition	NO_3_^-^	NH_4_^+^	NO_3_^-^	NH_4_^+^	NO_3_^-^	NH_4_^+^	NO_3_^-^	NH_4_^+^
	kg N ha^-1^ yr^-1^	μg N g soil^-1^		μg N g soil^-1^		μg N g soil^-1^		μg N g soil^-1^	
WI	0	2.59 (0.10)^a^	6.65 (0.66)^a^	2.08 (0.48)^a^	6.06 (1.05)^a^	1.49 (0.26)^a^	4.85 (0.50)^a^	1.54 (0.28)^a^	3.53 (0.23)^a^
	56	3.66 (0.38)^a^	7.02 (0.47)^a^	4.01 (0.31) ^a^	5.38 (0.44)^a^	1.99 (0.20)^a^^,b^	6.27 (0.68)^a^	2.43 (0.52)^a^	4.39 (0.58)^a^
	196	3.29 (0.26)^a^	5.31 (0.24)^a^	43.6 (16.7)^b^	9.39 (3.63)^a^	12.7 (8.67)^b^	6.38 (0.37)^a^	3.44 (0.53)^a^	3.32 (0.18)^a^
MI	0	2.44 (0.65)^a^	3.77 (0.52)^a^	2.01 (0.13)^a^	4.13 (0.48)^a^	1.07 (0.23)^a^	4.19 (0.63)^a^	1.20 (0.29)^a^	3.26 (0.32)^a^
	56	3.13 (0.11)^a^	3.21 (0.82)^a^	8.70 (0.94)^a^^,b^	4.77 (0.65)^a^	2.93 (1.56)^a^	3.64 (0.20)^a^	3.59 (0.79)^a^	2.64 (0.12)^a^
	196	1.80 (0.49)^a^	2.34 (0.51)^a^	22.6 (8.74)^b^	54.5 (11.04)^b^	3.20 (0.86)^a^	2.87 (0.22)^a^	10.6 (1.36)^b^	2.47 (0.41)^a^

^a^The mean of 4 replicates (standard error) is shown. Significant differences (α<0.05, determined by ANOVA) are indicated by different superscript letters within columns.

Total soil C and N did not vary among treatments within either site (ANOVA, all F<0.9, all p>0.4), but soil C (2.0 ± 0.09% C) and N (0.19 ± 0.009% N) at WI were nearly twice as high as at MI (0.85 ± 0.08% C and 0.08 ± 0.01% N). The C:N ratios were similar, with an average C:N ratio at WI of 10.6 ± 0.4 and an average C:N ratio of 10.8 ± 0.8 at MI.

### Root nitrogen fixation

**Fig 1:**
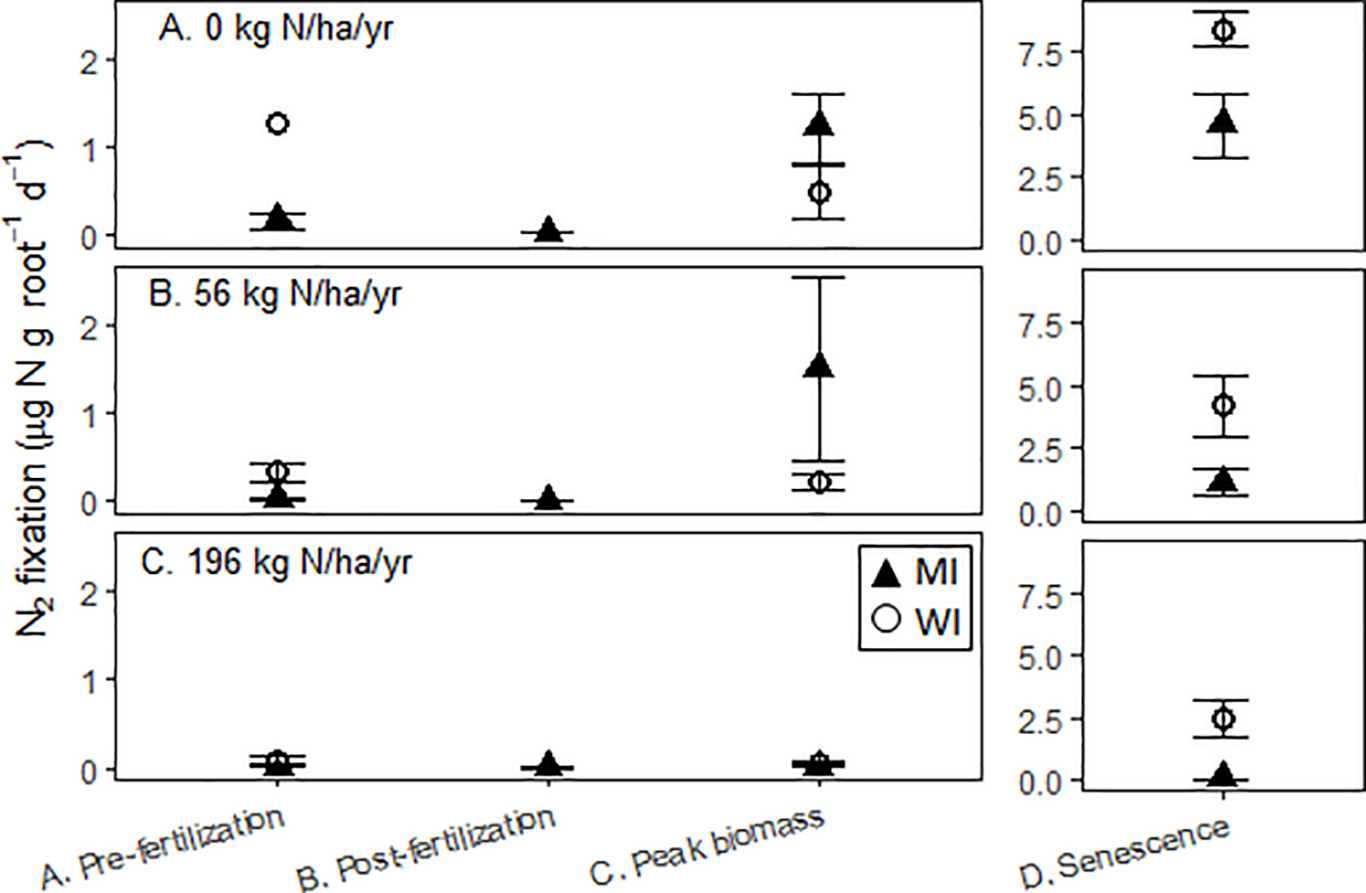
Potential switchgrass root ANF rates from two sites (MI: Kellogg Biological Station in Michigan, USA and WI: Arlington Agricultural Research Station, Wisconsin, USA) across four time periods. Roots were sampled from plots with a range of N fertilizer addition: A. unfertilized, B. 56 kg N ha^-1^ yr^-1^, and C. 196 kg N ha^-1^ yr^-1^. Note that the senescence period has a different scale to accommodate the much higher rates during that time period.

At the MI site, root ANF rates varied with season and N addition level, and the interaction term was also significant (adjusted r^2^ = 0.69, p<0.0001). More specifically, ANF rates decreased with N addition level and increased throughout the growing season, with the highest rates unexpectedly occurring after plant senescence (Fig 1). Each time period was statistically distinct (p<0.05), with post-fertilizer < pre-fertilizer < peak biomass < post-senescence (Fig 1). Rates ranged multiple orders of magnitude, from a minimum of 0.003 ± 0.003 to 4.6 ± 1.3 μg N g root^-1^ d^-1^.

At WI, root ANF also varied with season and N addition level (adjusted r^2^ = 0.88, p< 0.0001), but the interaction term was not significant. The highest rates occurred at post-senescence, with the other time periods not statistically different from one another (p>0.05, Fig 1). Rates ranged dramatically, from 0.06 ± 0.01 to 8.5 ± 0.69 μg N g root^-1^ d^-1^ (Fig 1).

In all seasons, root ANF rates declined with N addition level. At MI, the decline was exponential in all time periods, except peak biomass, when it declined linearly (S4 Fig). Similarly, at WI, the decline was exponential except during the post-senescence time period, when it was a linear decline (S4 Fig).

At WI, root ANF rates were partly explained by the soil N content, but not at MI. At WI, ANF decreased linearly as soil NH_4_^+^ concentration increased (r^2^ = 0.378, p = 0.0004) and decreased at an exponential rate with net mineralization (r^2^ = 0.463, p = 0.0005). At MI, soil N concentration was not significantly related to root ANF rate, irrespective of whether soil N was expressed as total inorganic N, NH_4_^+^, NO_3_^-^, or net mineralization rate.

### Soil nitrogen fixation rates

**Fig 2:**
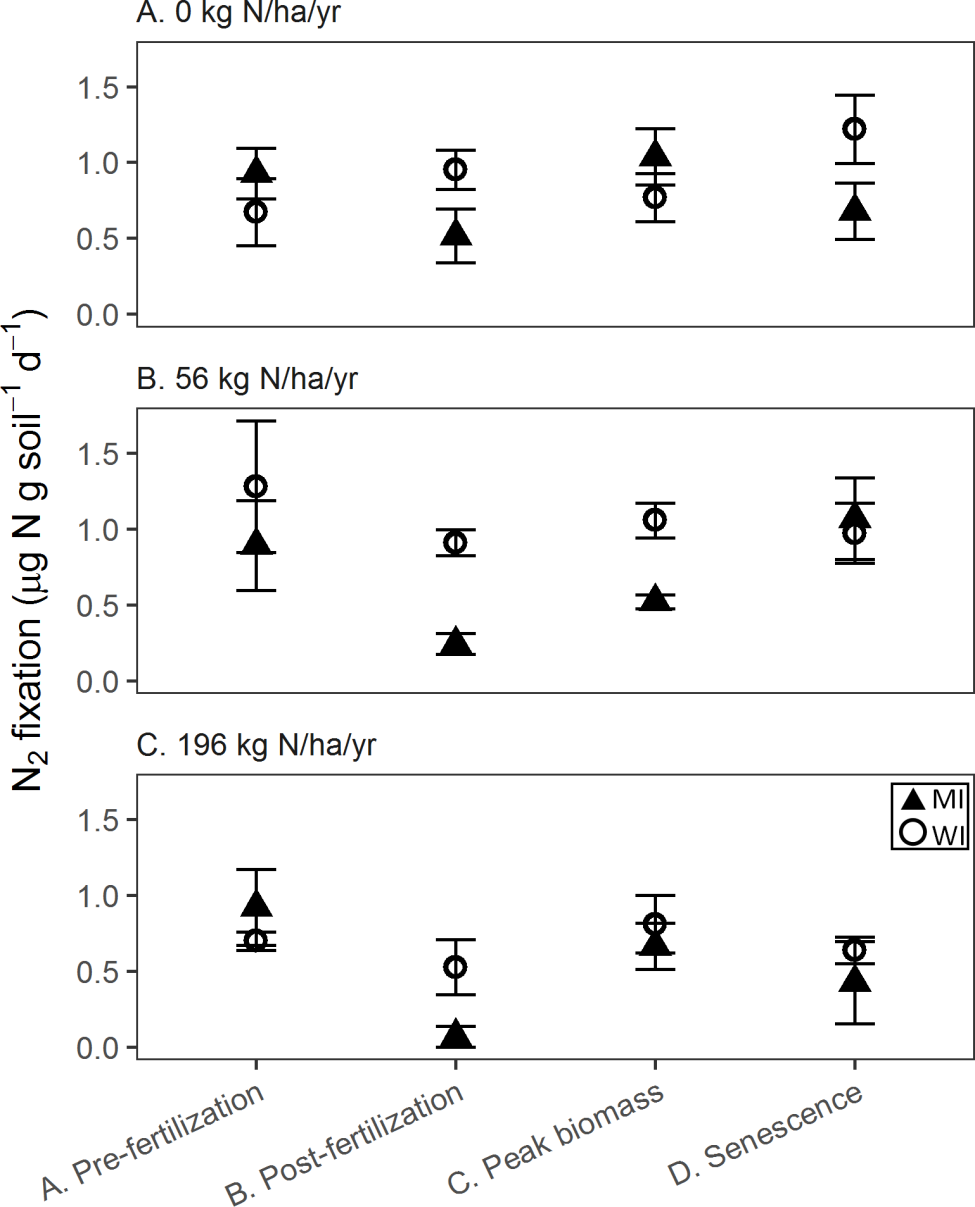
Soil potential ANF rates from two sites (MI: Kellogg Biological Station in Michigan, USA and WI: Arlington Agricultural Research Station, Wisconsin, USA) across four time periods. Soils were sampled from switchgrass rhizospheres grown with a range of N fertilizer addition: A. unfertilized, B. 56 kg N ha^-1^ yr^-1^, and C. 196 kg N ha^-1^ yr^-1^.

Soil ANF rates varied less dramatically than did the root fixation rates. At MI, rates ranged from a minimum rate of 0.07 ± 0.07 SE μg N g soil d^-1^ (with some replicates below detection) to a maximum rate of 1.07 ± 0.27 μg N g soil d^-1^ (Fig 2), with both time of sampling and N treatment predicting these rates (adjusted r^2^ = 0.31, p<0.001). In general, rates were lower in the N-fertilized plots and during the post-fertilizer time period (Fig 2). At WI, soil ANF had a higher minimum rate than at MI (0.53 ± 0.18 μg g soil d^-1^), but a similar maximum rate (1.22 ± 0.23 μg g soil d^-1^, Fig 2). Nitrogen treatment was the only significant predictor of soil ANF rates at WI, with lower rates in the fertilized plots, but it had little predictive power (adjusted r^2^ = 0.07, p<0.05).

More specifically, MI soil ANF declined with N addition post-fertilization and during peak plant biomass, but there were no differences among N treatments in the other sampling periods (S5 Fig). In both cases, ANF declined by a power function, with the highest rate in the unfertilized plots and equivalent, low ANF rates in both fertilized plots (S5 Fig). In WI, there was a linear decline in ANF rates with fertilizer addition during the post-fertilizer and post-senescence periods, but no significant differences among fertilizer levels during pre-fertilizer addition and peak biomass (S5 Fig).

In addition, soil ANF was inversely correlated with soil inorganic N content (not shown). At MI, soil ANF rates decreased linearly with total soil inorganic N (r^2^ = 0.31, p<0.0001), and with soil NO_3_^-^ content (r^2^ = 0.21, p = 0.01). The patterns were similar at WI, although soil N explained less of the variation in ANF (total soil inorganic N, r^2^ = 0.12, p = 0.017; soil NO_3_^-^ content r^2^ = 0.15, p = 0.0008). At both MI and WI, net mineralization rate and soil NH_4_^+^ concentration were not significant predictors of ANF.

### Annual nitrogen fixation

In extrapolating from our measured soil ANF rates, we estimated soil rhizosphere fixation from the unfertilized plots as 47 ± 7.5 kg N ha^-1^ yr^-1^ at MI and 39 ± 3.1 kg N ha^-1^ yr^-1^ at WI, which in good agreement with our mass balance estimates of 35 kg N ha^-1^ yr^-1^ (average of all years) to 58 kg N ha^-1^ yr^-1^ (2013–2015 average). In roots, we calculated annual fixation at MI as 0.71 ± 0.20 kg N ha^-1^ yr^-1^ in the unfertilized plots.

### Nitrogen mineralization rates

**Fig 3:**
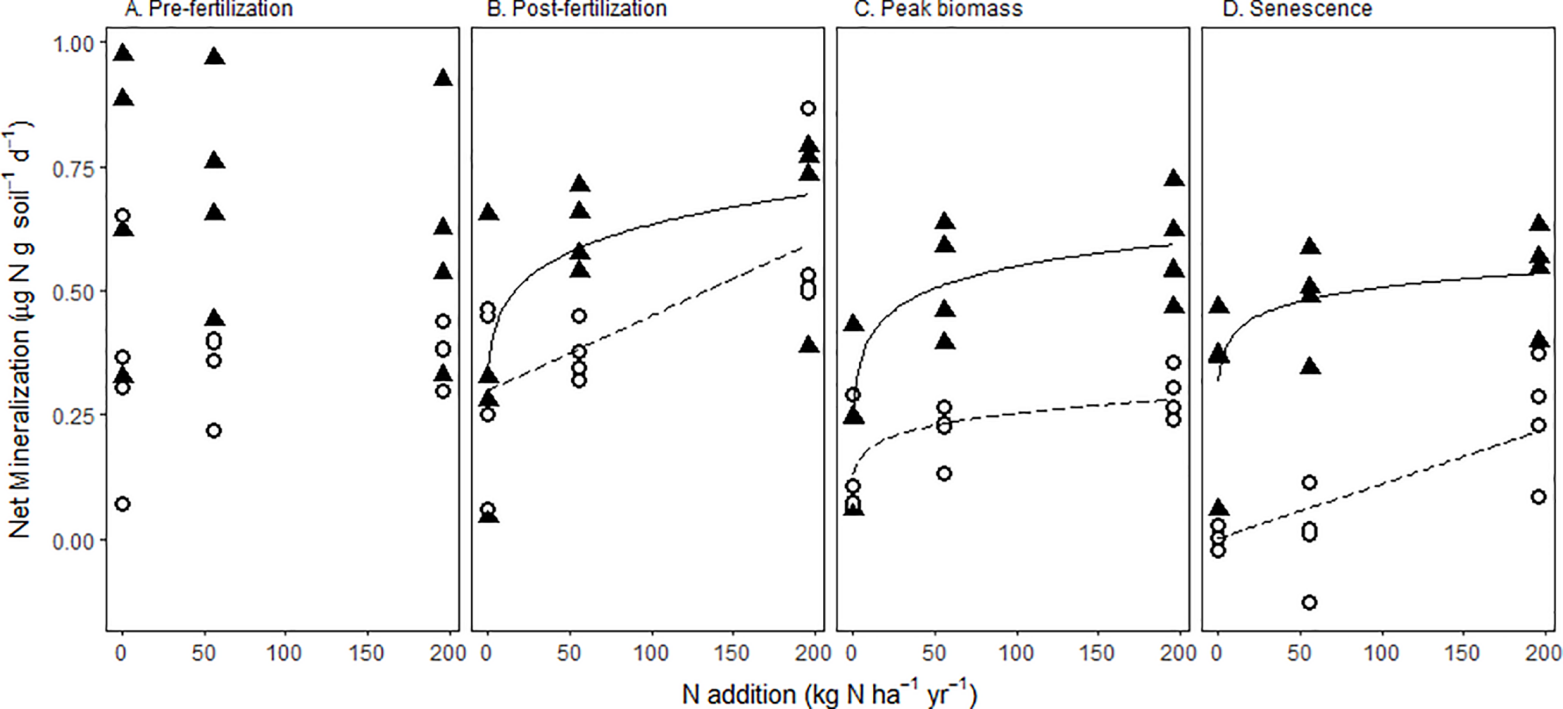
Net N mineralization rates, measured via 28-d aerobic incubations, during four time periods. Best-fit regression lines are shown where slopes were significantly different from 0. Soils were sampled from switchgrass rhizospheres at two sites (MI: Kellogg Biological Station, Michigan, USA and WI: Arlington Agricultural Research Station, Wisconsin, USA) and 3 fertilizer levels (unfertilized, 56 kg N ha^-1^ yr^-1^, and 196 kg N ha^-1^ yr^-1^). MI Post-fertilizer: y = 0.40*(x+1)^0.14, p = 0.04; MI Peak biomass: y = 0.07*log(x+1)+0.25, p = 0.002; MI Senescence: y = 0.04*log(x+1)+0.31, p = 0.03; WI Post-fertilizer: y = 0.002*x+0.299, p<0.02; WI Peak biomass: y = 0.127*(x+1)^0.15, p = 0.03; WI Senescence: y = 0.0012*x-0.015, p = 0.004.

Net N mineralization rates were influenced by site, season, and N level (r^2^ = 0.53, p<0.00001), but the interactions added no further predictive power. In general, N mineralization rates were higher at MI than at WI, with rates at MI ranging from 0.25 ± 0.08 to 0.70 ± 0.11 μg g soil^-1^ d^-1^. At WI, rates ranged from 0.002 ± 0.01 to 0.60 ± 0.09 μg g soil^-1^ d^-1^. Seasonal changes were modest, with the exception of the post-fertilizer addition period, when the fertilized plots at WI exhibited their highest net N mineralization (Fig 3). In general, N mineralization rates increased with N level, but only in the periods following fertilizer application. During those periods, the MI site exhibited a saturating relationship between N addition and net mineralization rate, and the WI site exhibited a linear relationship, except during peak biomass when it was saturating (Fig 3).

Net nitrification showed similar patterns, with a saturating relationship at MI and a linear relationship at WI between N level and net nitrification (S6 Fig). One deviation from this pattern occurred in the post-fertilizer addition period at MI, where rates increased linearly. The nitrification rates were almost always higher than mineralization rates, meaning that most of the inorganic N accumulated as NO_3_^-^. Relative nitrification, the percent of mineralizable N present as NO_3_^-^ at the end of the incubation [30, 45], generally increased with N addition. At MI, relative nitrification was >80% during all sampling periods, and increased to nearly 100% in the fertilized plots during all sampling periods. At WI, there were strong seasonal differences, with relative nitrification close to 100% prior to N addition and decreasing thereafter.

## Discussion

### Seasonal patterns in nitrogen fixation

We had hypothesized that the timing of ANF rates would mirror plant N demand (i.e., rates peaking at the height of the growing season when switchgrass N uptake is greatest), as has been observed in other non-nodulating associations [26, 46, 47]. Instead, we found that root ANF in the unfertilized plots was greatest by a factor of two at post-senescence (Fig 1). This may be in response to a surge in available post-senescence C. A similar pattern was found for dead root cells of a Patagonian conifer (*Lepidothamnus fonkii*) [48] and may illustrate an important, previously unrecognized feature of BNF: post-senescence ANF can bring N into the rhizosphere at a time when plant N uptake is nil. At the systems scale, these inputs can nevertheless be significant N sources.

Soil ANF rates were influenced by season at MI, but not at WI, with rates corresponding inversely to seasonal changes in N availability (Fig 2). However, we did not observe increased post-senescence ANF, as we did for roots, perhaps because soil diazotrophs are not as exposed to senescing root C. Soil ANF rates remained within a narrower range than root ANF. This narrow range, and the lack of seasonal patterns at WI, may be in part because the laboratory additions of C and water dampened the seasonal fluctuations in soil.

### Effect of N addition on nitrogen fixation

Both soils and roots generally exhibited a decline in ANF rates with N addition, but the patterns were more consistent and pronounced in the roots. The sensitivity of roots to N availability and plant phenology may stem from the physical proximity of root microbes to the zone of depleted N and to C-rich root exudates. Interestingly, while root ANF rates were consistently related to N fertilizer treatment, measurements of soil inorganic N concentration and mineralization usually did not correlate with root ANF rates. This may be because the high degree of seasonal variation (i.e., high post-senescence ANF) overrode the effects of N availability.

It may also be that the legacy of long-term N addition is more important in determining root ANF rates than is instantaneous N availability. Prior to fertilizer addition (“pre-fertilization”, Fig 1), the 0-N plots had higher root ANF rates than the 56-N and 196-N plots, even though soil N mineralization rates and inorganic N concentrations were equivalent among the treatments. One potential explanation for these higher rates–an apparent legacy effect–is a diazotroph community better primed for taking advantage of instantaneous C inputs from roots. This potential mechanism is supported by others’ observations that long-term N fertilizer additions can influence microbial community structure [49, 50]. Perhaps a switchgrass environment with small or no N inputs is likely to favor diazotrophs, allowing for high rates of N fixation when C is abundant (i.e., during root decomposition or exudation).

In contrast, soil ANF had a muted response to N addition, but rates were negatively correlated with inorganic N concentrations. Thus, in soils, ANF appears to be controlled by short-term (i.e., seasonal) changes in N availability, while in the roots, ANF may be controlled by the long-term effects of N addition.

### Nitrogen fixation measurements in context

Our range of measured soil ANF rates was narrower than measurements in arid grassland, but our range of root ANF rates was wider. Using the same method as the present study, Gupta et al. [35] measured soil N_2_ fixation rates of 0.90 to 2.35 μg N g^-1^ d^-1^ beneath Australian pasture grasses (our range: 0.07 to 1.22 μg N g^-1^ d^-1^). We measured a wider range of rates in roots than did previous studies, with Gupta et al. [35] measuring 0.75 to 1.25 μg N g root^-1^ d^-1^ in Australian pasture grasses and Morris et al. [19] measuring 0.29 to 0.49 μg N g root^-1^ d^-1^ in intact soil cores collected from Texas, USA prairies (our range: 0.003 to 4.6 μg N g root^-1^ d^-1^). These studies occurred at single time points during peak growing season, however, and did not incorporate the post-senescence time period.

Our annual estimates produced comparable ANF rates in roots and higher rates in soil compared to previous switchgrass ANF studies. Previously, root ANF rates were estimated from 1.0 to 2.4 kg N ha^-1^ yr^-1^ in Texas prairies using ^15^N_2_ addition to intact soil cores [19], which is close to our estimate of 0.71 kg N ha^-1^ yr^-1^. In contrast, our annual soil ANF estimates were at least 10× higher than the previous estimate of ANF rates in soils beneath switchgrass (3.6 kg N ha^-1^ yr^-1^ in Wisconsin prairies, [20]; 39–47 kg N ha^-1^ yr^-1^ via extrapolation and 35–58 kg N ha^-1^ yr^-1^ via mass balance in this study). This discrepancy may arise from methodological differences; we used ^15^N_2_ incubations and Tjepkema and Burris [20] used the acetylene reduction assay. The two techniques often produce rates that are not consistent with one another [12, 19]. Alternatively, these two studies could represent the wide range of N_2_ fixation rates among sites. Additional measurements of ANF in temperate grasslands are warranted.

Globally, ANF has been estimated to range from 0 to 15 kg N ha^-1^ yr^-1^ and free-living N fixation from 1 to 10 kg N ha^-1^ yr^-1^ [12]. Both of these estimates are uncertain, however, because the scant number of ANF studies have methodological inconsistencies [13]. Some tropical grasses, particularly sugarcane, may fix as much as 40 kg N ha^-1^ yr^-1^ [51]. Our upper-bound estimates suggest that switchgrass (and, by extension, other temperate C4 perennial grasses) may receive ANF inputs that are lower than tropical inputs but that exceed estimated global averages.

ANF measurements are relatively rare for temperate grasslands, but we hypothesize that other temperate prairie grasses grown under low-N inputs will display similar patterns to those documented here. Indeed, some restored prairies are non-responsive to N inputs [52, 53], suggesting that plant-microbe processes are well-equilibrated to low-N inputs [54], perhaps in part because of ANF contributions.

### Internal nitrogen cycling dynamics

Net mineralization and net nitrification rates generally increased with N addition at both sites, consistent with previous studies [28, 55]. In nearly all plots and sampling periods, net nitrification rates exceeded net mineralization rates; that is, most of the inorganic N accumulated as NO_3_^-^ rather than NH_4_^+^, a pattern similarly observed in both grassland [56] and forest ecosystems [29]. Thus, the relationship between soil N and internal N cycling rates beneath switchgrass conformed to expectations. More importantly, the increase in mineralization with N addition demonstrated that switchgrass was not compensating for low N inputs via stimulation of mineralization. In fact, unfertilized soils likely experienced greater microbial immobilization of N (i.e., net nitrification > net mineralization, Fig 3, S6 Fig), contributing to even lower N availability at low N fertilizer levels.

### Mass balance suggests substantial nitrogen inputs from fixation

The unfertilized switchgrass plots at MI had a cumulative N deficit of 247 kg N ha^-1^ over the 7-year course of the experiment, or ~35 kg N ha^-1^ yr^-1^, on average. This is almost certainly an underestimate, because our mass balance calculation does not include N lost by leaching (approximately 2.7 kg N ha^-1^ yr^-1^, [18]) or from denitrification. While it is possible that some of this N might be supplied through net mineralization of soil organic N stores, especially since switchgrass and other perennial grasses are adept at recovering N from soils through their association with mycorrhizal fungi [57, 58], we have seen no evidence of soil N depletion at this site [18, 59], consistent with long-term gains in soil organic matter commonly observed when cultivated soils are converted from annual to perennial vegetation (e.g., [60, 61]). The total N deficit is equivalent to ~7% of the total soil N pool, which is within detection limits if soil organic matter were the sole source of the missing N [62]. Soil organic matter is unlikely to be the sole source of N; soil organic carbon remained unchanged from 2008–2013 and N fertilizer addition had no effect on soil organic carbon concentrations at this site [18].

This cumulative N deficit accounts for inputs from N deposition (average of 6.2 kg N ha^-1^ yr^-1^), leaving an average deficit of 35.2 kg N ha^-1^ yr^-1^. The N deficit has increased as the switchgrass established; the post-establishment phase (2013–2015) average N deficit was 57.8 kg N ha^-1^ yr^-1^. Mass balance data are not available for the WI site, but the measured ANF rates suggest that the switchgrass rhizosphere there has the capacity to fix nearly as much N as at the MI site. While mass balance differences are not always a reliable measure of N fixation [63], if major inputs and outputs are few and well-characterized (in our case, deposition N and harvest N, respectively), N fixation by difference can be estimated with confidence, so long as soil organic matter is not declining, which it is not [18]. Furthermore, extrapolation of lab measurements (47 kg N ha^-1^ yr^-1^ at MI) provides good agreement with mass balance results, and suggests that ANF can make up the N mass balance deficit.

### Site comparisons

Our two study sites have similar climatic conditions, but markedly different soils, with WI mollisols containing more total C and total N than MI alfisols. Soils from the WI site generally had lower net N mineralization rates than the MI site, though, suggesting that its soil N is less available to plants, presumably due to immobilization in microbial biomass or soil organic matter. In addition, soils at WI have a finer texture (more clay) with a larger number of reactive surfaces and greater potential for chemical and mineral immobilization. The WI site usually had slightly higher soil and root ANF rates, perhaps reflective of greater N demand caused by its lower N mineralization rates. In addition, soils at WI contained more C, perhaps indicating more microbial biomass and greater C availability for N_2_ fixers.

### Conclusions and hypotheses

As we hypothesized, ANF decreased with N addition; the unfertilized plots generally had the highest ANF rates. We had further expected that ANF rates would rise during the growing season, as plant N demand increases. Instead, we observed minimal seasonal changes in soil N fixation, and root ANF was asynchronous with plant N demand, with the highest ANF occurring after the plants senesced. Based on our lab measurements and mass balance calculations, we further hypothesize that ANF appears to be an important N input in unfertilized perennial temperate grasslands. If ANF inputs are important to grassland ecosystems, our results suggest that they occur throughout and beyond the growing season, at times asynchronous with plant N demand. As such, future attempts to estimate annual ANF inputs should employ readily-scalable techniques, such as in situ or whole-plant ^15^N_2_ incubations, and measure ANF frequently throughout the year.

Data are available from the Dryad Digital Repository [64] at doi:10.5061/dryad.60bn81v.

## Supporting information

S1 Text Assessing effects of laboratory conditions on N_2_ fixation estimates. (DOCX)

S2 Text Contamination checks on ^15^N_2_. (DOCX)

S1 Fig Fixation rates in soils with the addition of glucose solution (+Glucose) and with the addition of deionized water (-Glucose). All samples were at 100% water-filled pore space. They were sampled from the WI site, during the pre-fertilizer time period. Fixation was measured with the ^15^N_2_ incubation method.
(TIFF)

S2 Fig Effects of glucose, headspace oxygen, and soil moisture on soil fixation rates. Soil fixation was measured with the acetylene reduction assay and fixation is expressed as nmol of ethylene accumulated. Abbreviations are as follows: +C = glucose added as a solution of 4% glucose, to 100% water-filled pore space; 0 C = deionized water added, to 100% water-filled pore space; p C = powdered glucose added, to achieve the same C concentration as would be achieved with +C; 4% O_2_ = headspace adjusted to 4% O_2_ at beginning of incubation; 20% O_2_ = headspace adjusted to 20% O_2_ at beginning of incubation.
(TIFF)

S3 Fig Effects of glucose concentration on root fixation. Root fixation was measured with the acetylene reduction assay and fixation is expressed as nmol of ethylene accumulated. Abbreviations are as follows: DI = deionized water, roots incubated without any added carbon; Low C = roots incubated with 1.6 mg C g root^-1^; Mid C = roots incubated with 3.2 mg C g root^-1^; High C = roots incubated with 16 mg C g root^-1^.
(TIFF)

S4 Fig N_2_ fixation on roots by N fertilizer addition across four time periods. Best-fit regression lines are shown where slopes were significantly different from 0. Roots were sampled from switchgrass rhizospheres at two sites (MI: Kellogg Biological Station, Michigan, USA and WI: Arlington Agricultural Research Station, Wisconsin, USA) and 3 fertilizer levels (unfertilized, 56 kg N ha^-1^ yr^-1^, and 196 kg N ha^-1^ yr^-1^). MI Pre-fertilizer: y = 0.13*e^((x+1)*-0.01), MI Post-fertilizer: y = 0.04*(x+1)^-0.59, MI Peak biomass: y = 1.67–0.01*x, MI Senescence: y = 4.75*e^((x+1)*-0.03), WI Pre-fertilizer: y = 0.90*e^((x+1)*-0.01), WI Peak biomass: y = 0.45*e^((x+1)*-0.01), WI Senescence: y = 7.2–0.03*x.
(TIF)

S5 Fig N_2_ fixation in soil by N fertilizer addition across four time periods. Best-fit regression lines are shown where slopes were significantly different from 0. Soils were sampled from switchgrass rhizospheres at two sites (MI: Kellogg Biological Station, Michigan, USA and WI: Arlington Agricultural Research Station, Wisconsin, USA) and 3 fertilizer levels (unfertilized, 56 kg N ha^-1^ yr^-1^, and 196 kg N ha^-1^ yr^-1^). MI Post-fertilizer: y = 0.54*(x+1)^-0.26, MI Peak biomass: y = 1.02*(x+1)^-0.11, WI Post-fertilizer: y = 0.99–0.0023*x, WI Senescence: y = 1.19–0.003*x.
(TIFF)

S6 Fig Net nitrification rates, measured via 28-d aerobic incubations, during 4 time periods. Best-fit regression lines are shown where slopes were significantly different from 0. Soils were sampled from switchgrass rhizospheres at two sites (MI: Kellogg Biological Station, Michigan, USA and WI: Arlington Agricultural Research Station, Wisconsin, USA) and 3 fertilizer levels (unfertilized, 56 kg N ha^-1^ yr^-1^, and 196 kg N ha^-1^ yr^-1^). Best-fit regression lines are shown where slopes were significantly different from 0. MI Post-fertilizer: y = 0.08*x+0.34, MI Peak biomass: y = 0.06*log(x+1)+0.35, MI Senescence: y = 0.04*log(x+1)+0.38, WI Post-fertilizer: y = 0.002*x-0.312, WI Peak biomass: y = 0.001*x+0.25, WI Senescence: y = 0.001*x+0.02.
(TIFF)
