# Supplementary material for: Associative nitrogen fixation (ANF) in switchgrass (Panicum virgatum) across a nitrogen input gradient
Source: PLoS One. 2018 Jun 1;13(6):e0197320. doi: 10.1371/journal.pone.0197320 (PMC5983442; doi:10.1371/journal.pone.0197320)
Supplement: S1 Text — (DOCX) [file pone.0197320.s001.docx]

**S1 Text. Assessing effects of laboratory conditions on N_2_ fixation estimates**

Laboratory N_2_ fixation potential assays are easier and less expensive than whole-plant or field assays, which allow for more treatments and replicates, but they create some artifacts. Our method may inflate the N_2_ fixation estimates in three ways: 1) glucose addition, 2) low oxygen concentration, and 3) diazotroph population growth over the 7-d incubation. High C availability and low oxygen both promote N_2_ fixation [1], and an environment optimized for N_2_ fixation may favor the growth of diazotrophs. We thus conducted a series of experiments to quantify the effect of these conditions on our rate estimates.

To determine the effect of glucose addition on our soil ANF rate estimates, we incubated a third set of vials, to which we added deionized water instead of glucose solution. Those vials received the same amount of ^15^N_2_ gas as the glucose-amended vials, and underwent the same ^15^N_2_ incubation and calculation procedures as described in the main text.

We further assessed laboratory effects, by manipulating C, headspace oxygen concentration, and soil moisture. For this set of experiments, we used the acetylene reduction assay (ARA, described in detail below). We added 5g of soil to the vials and then added either: 1) 4% glucose solution, 2) deionized water, or 3) powdered glucose to each of 10 vials. The liquids were added to bring WFPS to 100% and the powdered glucose was added to achieve the same total C as the glucose solution. We mixed the powdered glucose into the soil before adding it to the vial. We evacuated each vial, then added 16 ml (glucose and water treatments) or 18 ml (powdered glucose treatment) of an artificial atmosphere. Five replicates of each C treatment received low O_2_ (4%) and five replicates of each C treatment received ambient O_2_ (20%). The low O_2_ treatment corresponds to the average % O_2_ in the headspace at the conclusion of our ^15^N_2_ incubations. Other components of the artificial atmosphere were N_2_ (70%), acetylene (10%), and helium (low oxygen treatment only; 16%). We then measured N_2_ fixation with ARA, as described below.

We completed similar experiments on roots. We incubated switchgrass roots at 4 different glucose levels: 0, 1.6, 3.2, and 16 mg C g root^-1^. The range of C added to roots in the experiment was 0.6- 3.2 mg C g root^-1^; with this experiment, we covered that range, along with both higher and lower concentrations. We did not assess the effect of O_2_ concentration on roots, because all the root incubations were aerobic; the roots were not submerged and headspace remained oxygenated for the entire incubation. The volume of glucose solution added (0.08-0.2 ml) was not sufficient to keep the roots in a standing solution; the entire volume of the Exetainer was headspace and root biomass.

We used the ARA [2, 3], which assesses the activity of the nitrogenase gene. Nitrogenase will reduce acetylene to ethylene, and so samples are incubated with acetylene, and the ethylene accumulation rate is used as a metric of nitrogenase activity. We generated acetylene in the lab by adding deionized water to calcium carbide. We added 10% acetylene to each vial and incubated it for 24 h. We then sampled the headspace and measured the ethylene concentration on an Agilent 7890A gas chromatograph (Wilmington, DE, USA) equipped with a flame ionization detector. The samples ran through a 3 m Porapak N 80/100 column, through which we achieved clear separation of the ethylene and acetylene peaks. To account for any other sources of ethylene (e.g., from biological activity or from contamination), we measured ethylene in the gas mixture added to each vial, as well as in vials incubated without acetylene (i.e., no-acetylene controls). We detected no ethylene in the no-acetylene controls, but we did detect small quantities of ethylene in the gas mixture, which we subtracted from our ethylene production.

To calculate fixation, we first calculated the ethylene accumulation rate as the ethylene concentration after 24 h, minus ethylene present in the added acetylene, divided by the length of the incubation. We then used the ideal gas law to convert this rate to a molar rate, and divided by the dry weight of the soil or root to get nitrogenase activity in nmol ethylene/g/h.

*Results*

The addition of glucose to soils significantly increased the N_2_ fixation rates (paired t-test, t=4.9, p=0.0003). In the absence of glucose, soil N_2_ fixation in the 0-N plots was 0.14 ± 0.12 μg N g soil^-1^ d^-1^, but with glucose, soil N_2_ fixation was 0.65 ± 0.17 μg N g soil^-1^ d^-1^ (S1 Fig). The addition of glucose solution thus boosted N_2_ fixation rates in unfertilized soils by a factor of 4.8, on average.

Results from the ARA assay revealed that both water and C are required to stimulate N_2_ fixation in soils (S2 Fig). Neither additions of C alone (added as powdered glucose) nor water alone (added as DI water to 100% WFPS) increased N_2_ fixation rates (ANOVA with Tukey’s HSD, all pairwise comparisons p>0.8). Alterations to headspace O_2_ concentration similarly had no effect (ANOVA with Tukey’s HSD, all pairwise comparisons p>0.2). Rates increased only when glucose was added as a solution (ANOVA with Tukey’s HSD, p< 4E-7).

The addition of glucose to roots did not increase the N_2_ fixation rates; in fact, the highest rates occurred when DI water alone was added, while the different levels of C addition were indistinguishable from one another (ANOVA with Tukey’s HSD, water alone > high C, p=0.018, water alone > low C, p =0.015, all other comparisons p>0.1; S3 Fig).

*References*

1. Robson RL, Postgate JR (1980) Oxygen and hydrogen in biological nitrogen fixation. Annual Review of Microbiology 34: 183-207. doi: 10.1146/annurev.mi.34.100180.001151.
2. Myrold DD, Ruess RW, Klug MI (1999) Dinitrogen fixation. In: GP Robertson, DC Coleman, CS Bledsoe, P Sollins (eds) Standard Soil Methods for Long-Term Ecological Research. Oxford University Press, New York, New York, USA.
3. Weaver RW, Danso SKA (1994) Dinitrogen fixation. In: RW Weaver, JS Angle, PJ Bottomley (eds) Methods of Soil Analysis. American Society of Agronomy, Madison, WI, USA.
