# Supplementary material for: Associative nitrogen fixation (ANF) in switchgrass (Panicum virgatum) across a nitrogen input gradient
Source: PLoS One. 2018 Jun 1;13(6):e0197320. doi: 10.1371/journal.pone.0197320 (PMC5983442; doi:10.1371/journal.pone.0197320)
Supplement: S2 Text — (DOCX) [file pone.0197320.s002.docx]

**S2 Text. Contamination checks on ^15^N_2_**

Commercially-available ^15^N_2_ can be contaminated with other forms of reactive ^15^N, including ^15^NH_3_ and ^15^NO_x_^-^, that readily oxidize to ^15^NO_3_^-^ when dissolved in water (Dabundo, Lehmann et al. 2014). These N forms are readily taken up by plants and microbes, which can result in ^15^N enrichment in the absence of fixation and thus inflate BNF estimates or result in false positives. We are confident that contamination did not influence our results. We tested for contamination by incubating our source ^15^N_2_ (from Cambridge Isotope Laboratories, Tewksbury, MA, USA) with non-N_2_-fixing diatoms (*Cyclotella*) in a sealed bottle for 24 hours with ^15^N_2_ and found no difference in ^15^N content, compared to a control incubated with lab air. In addition, a simple calculation shows that the potential contamination is unlikely to affect our results: If our gas had been contaminated at the highest level reported for Cambridge Isotopes gas by Dabundo et al. [1], and we assume that all contaminants were incorporated into soil, the soil would have 0.0000003 atom% more ^15^N than a control, which translates to an average N_2_ fixation rate of 0.000019 ± 0.0000002 μg N g soil^-1^ d^-1^. This is less than our detection limit, and if subtracted from the measured rates, would have a negligible effect on reported rates. If we apply the same assumptions to the root data, the roots would accumulate 0.092 atom % ^15^N, which translates to 0.0008 ± 0.0003 μg N g root^-1^ d^-1^. Also note that there were numerous samples, particularly in the 196-N plots, where N_2_ fixation was not detectable in either roots or soils. If our results were inflated by contamination, then we would not have had any samples with undetectable N_2_ fixation.

*Reference*

1. Dabundo R, Lehmann MF, Treibergs L, Tobias CR, Altabet MA, Moisander PH, Granger J (2014) The Contamination of Commercial N-15(2) Gas Stocks with N-15-Labeled Nitrate and Ammonium and Consequences for Nitrogen Fixation Measurements. Plos One 9. doi: 10.1371/journal.pone.0110335.
