# Supplementary figures and images for: Associative nitrogen fixation (ANF) in switchgrass (Panicum virgatum) across a nitrogen input gradient
Source: PLoS One. 2018 Jun 1;13(6):e0197320. doi: 10.1371/journal.pone.0197320 (PMC5983442; doi:10.1371/journal.pone.0197320)

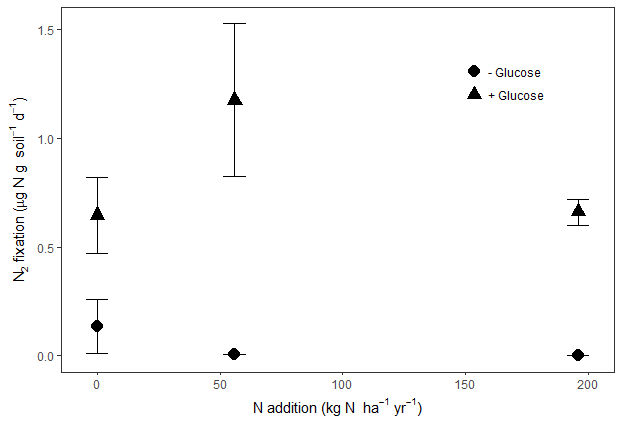

Supplement: S1 Fig — All samples were at 100% water-filled pore space. They were sampled from the WI site, during the pre-fertilizer time period. Fixation was measured with the 15N2 incubation method. (TIFF) [file pone.0197320.s003.tiff]

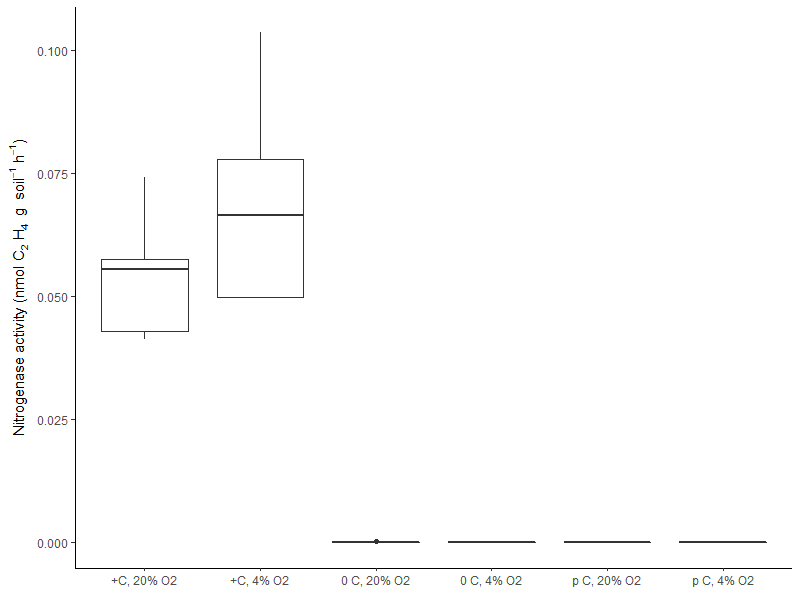

Supplement: S2 Fig — Soil fixation was measured with the acetylene reduction assay and fixation is expressed as nmol of ethylene accumulated. Abbreviations are as follows: +C = glucose added as a solution of 4% glucose, to 100% water-filled pore space; 0 C = deionized water added, to 100% water-filled pore space; p C = powdered glucose added, to achieve the same C concentration as would be achieved with +C; 4% O2 = headspace adjusted to 4% O2 at beginning of incubation; 20% O2 = headspace adjusted to 20% O2 at beginning of incubation. (TIFF) [file pone.0197320.s004.tiff]

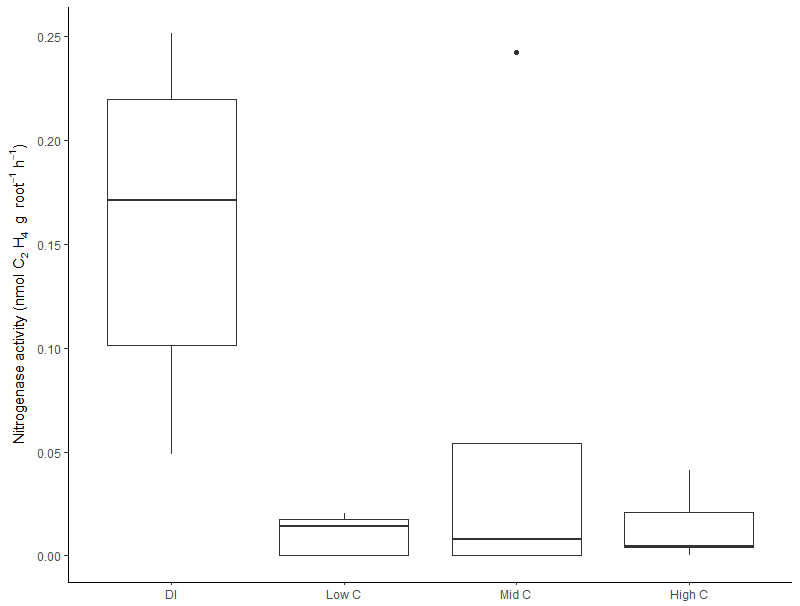

Supplement: S3 Fig — Root fixation was measured with the acetylene reduction assay and fixation is expressed as nmol of ethylene accumulated. Abbreviations are as follows: DI = deionized water, roots incubated without any added carbon; Low C = roots incubated with 1.6 mg C g root-1; Mid C = roots incubated with 3.2 mg C g root-1; High C = roots incubated with 16 mg C g root-1. (TIFF) [file pone.0197320.s005.tiff]

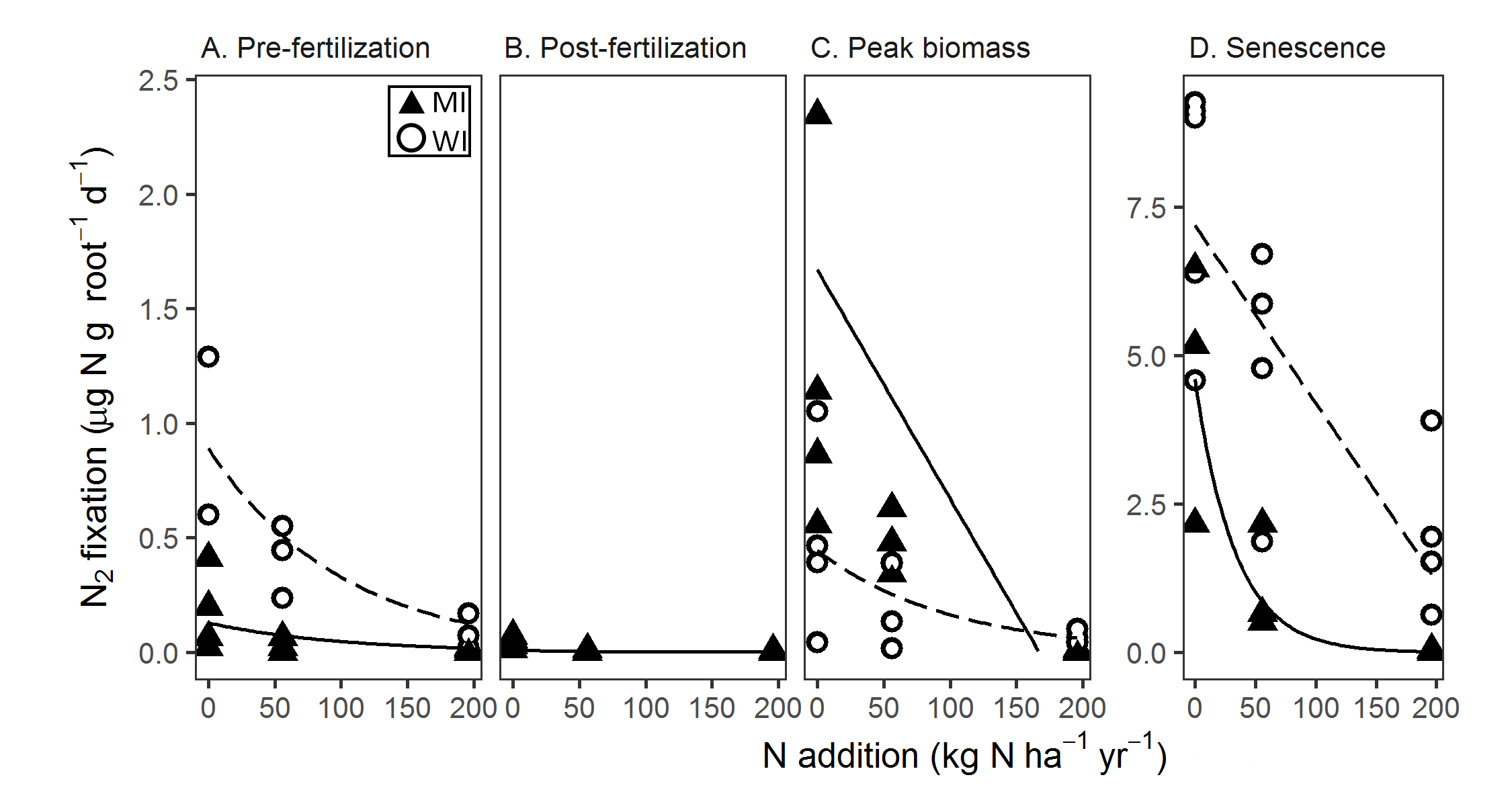

Supplement: S4 Fig — Best-fit regression lines are shown where slopes were significantly different from 0. Roots were sampled from switchgrass rhizospheres at two sites (MI: Kellogg Biological Station, Michigan, USA and WI: Arlington Agricultural Research Station, Wisconsin, USA) and 3 fertilizer levels (unfertilized, 56 kg N ha-1 yr-1, and 196 kg N ha-1 yr-1). MI Pre-fertilizer: y = 0.13*e^((x+1)*-0.01), MI Post-fertilizer: y = 0.04*(x+1)^-0.59, MI Peak biomass: y = 1.67–0.01*x, MI Senescence: y = 4.75*e^((x+1)*-0.03), WI Pre-fertilizer: y = 0.90*e^((x+1)*-0.01), WI Peak biomass: y = 0.45*e^((x+1)*-0.01), WI Senescence: y = 7.2–0.03*x. (TIF) [file pone.0197320.s006.tif]

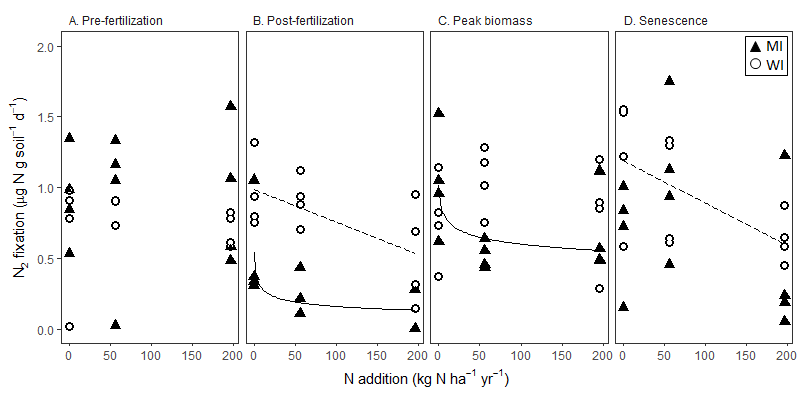

Supplement: S5 Fig — Best-fit regression lines are shown where slopes were significantly different from 0. Soils were sampled from switchgrass rhizospheres at two sites (MI: Kellogg Biological Station, Michigan, USA and WI: Arlington Agricultural Research Station, Wisconsin, USA) and 3 fertilizer levels (unfertilized, 56 kg N ha-1 yr-1, and 196 kg N ha-1 yr-1). MI Post-fertilizer: y = 0.54*(x+1)^-0.26, MI Peak biomass: y = 1.02*(x+1)^-0.11, WI Post-fertilizer: y = 0.99–0.0023*x, WI Senescence: y = 1.19–0.003*x. (TIFF) [file pone.0197320.s007.tiff]

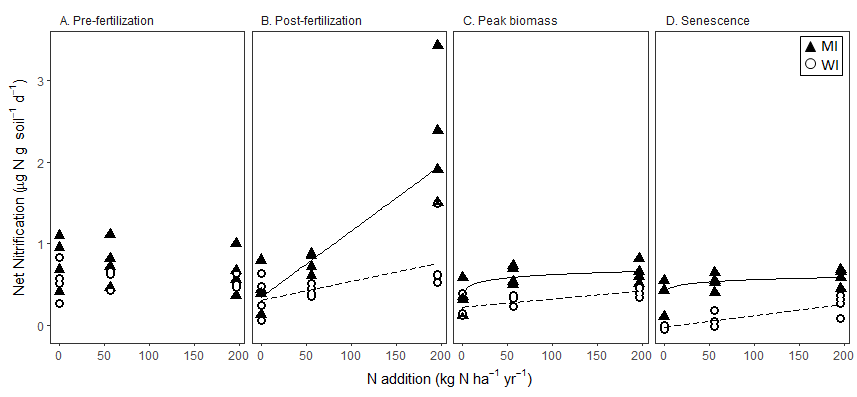

Supplement: S6 Fig — Best-fit regression lines are shown where slopes were significantly different from 0. Soils were sampled from switchgrass rhizospheres at two sites (MI: Kellogg Biological Station, Michigan, USA and WI: Arlington Agricultural Research Station, Wisconsin, USA) and 3 fertilizer levels (unfertilized, 56 kg N ha-1 yr-1, and 196 kg N ha-1 yr-1). Best-fit regression lines are shown where slopes were significantly different from 0. MI Post-fertilizer: y = 0.08*x+0.34, MI Peak biomass: y = 0.06*log(x+1)+0.35, MI Senescence: y = 0.04*log(x+1)+0.38, WI Post-fertilizer: y = 0.002*x-0.312, WI Peak biomass: y = 0.001*x+0.25, WI Senescence: y = 0.001*x+0.02. (TIFF) [file pone.0197320.s008.tiff]
